# Performance Analysis and Optimization of an InGaAs/GaAsSb Heterojunction Dopingless Tunnel FET with a Heterogate Dielectric

**DOI:** 10.3390/mi16121330

**Published:** 2025-11-26

**Authors:** JunJie Huang, HongXia Liu, Shupeng Chen, Shulong Wang, Chen Chong, Chang Liu

**Affiliations:** 1 Key Laboratory for Wide Band Gap Semiconductor Materials and Devices of Education, School of Microelectronics, Xidian University, Xi’an 710071, China; huangjunjie2024@163.com (J.H.); spchen@xidian.edu.cn (S.C.); slwang@xidian.edu.cn (S.W.); 18829029042@163.com (C.C.); xd_liuchang@163.com (C.L.); 2 Science and Technology on Reliability Physics and Application Technology of Electronic Component Laboratory, China Electronic Product Reliability and Environmental Testing Research Institute, Guangzhou 511370, China

**Keywords:** band-to-band tunneling, InGaAs/GaAsSb heterojunction, dopingless TFET, heterogate dielectric, DC and RF

## Abstract

An InGaAs/GaAsSb heterojunction dopingless Tunnel FET with a heterogate dielectric is proposed and investigated in this work, aiming to extend the advantages of dopingless TFETs in low-power applications. By employing the InGaAs/GaAsSb heterojunction with a quasi-broken gap energy band structure in dopingless TFET, the HDL-TFET achieves extremely high band-to-band tunneling efficiency. A dual-electrode structure is adopted to improve carrier distribution, which further enhances tunneling efficiency and increases on-state current (I_ON_). To suppress off-state tunneling, optimize ambipolar current, and reduce parasitic capacitance, a heterogate dielectric structure is introduced. Results show that the HDL-TFET exhibits an I_ON_ up to 8.33 × 10^−5^ A/μm and a steep subthreshold swing (SS_avg_) of 10.18 mV/dec at a low operating voltage of 0.5 V. It also achieves an off-state current (I_OFF_) as low as 3.42 × 10^−15^ A/μm and I_ON_/I_OFF_ ratio up to 2.44 × 10^10^, with no obvious ambipolar current. Compared with previously reported works, the proposed HDL-TFET demonstrates significant advantages. Additionally, the introduction of the heterogate dielectric and dual-electrode structure significantly improves the RF performance of the device, with a peak transconductance (G_m_) of 333 μS/μm, and a peak cutoff frequency (f_T_) and gain bandwidth product (GBP) up to 64 GHz and 49 GHz, respectively.

## 1. Introduction

Unlike traditional MOSFETs, TFETs primarily rely on the band-to-band tunneling (BTBT) mechanism for current conduction. This mechanism can effectively break the limitation of thermionic emission and achieve a subthreshold swing lower than 60 mV/dec, making TFET ideal logic devices for low-power applications [1,2,3,4]. To improve device performance, conventional TFETs require an extremely steep doping concentration gradient at the tunneling junction. This requirement leads to high thermal budgets during fabrication and performance degradation caused by random doping fluctuations [5,6]. To address these issues, dopingless TFETs have been extensively studied [7,8,9,10,11,12]. In dopingless TFET, the source and drain regions are formed based on the concept of charge plasma [13] by configuring different electrode work functions. This design avoids the high process requirements imposed by abrupt junctions and the impact of random doping fluctuations.

Another issue limiting TFET performance is the relatively low on-state current (I_ON_) [14,15]. Among semiconductor materials, III-V compounds exhibit great potential due to their small effective mass and broken or staggered band alignment. These properties enable higher I_ON_ and a more optimized I_ON_/I_OFF_ ratio [16,17]. To achieve better TFET performance, many researchers have conducted in-depth studies on heterojunction TFETs based on various III-V materials, such as GaSb/InAs [18,19,20], InGaAs/GaAsSb [21,22], InGaAs/InAlAs [23], and InGaAs/InP [24]. However, most of these studies focus on conventionally doped TFET, while research on dopingless TFETs remains relatively limited. Only Reference [25] has investigated a similar structure, but the proposed structure does not leverage the advantages of the quasi-broken gap energy band. Its tunneling junction is entirely composed of GaAsSb, and the device performance is improved through the material’s inherently small band gap—this operating mechanism is completely different from that of the device studied in this work. Moreover, the performance of the device studied in this work is further improved compared with that in Reference [25].

In this work, a dopingless TFET structure (HDL-TFET) based on the InGaAs/GaAsSb heterojunction is proposed, and its DC and RF performances are investigated. Benefiting from the quasi-broken gap energy band structure of the InGaAs/GaAsSb heterojunction and the process advantages of dopingless TFET, the HDL-TFET achieves both simple fabrication and large on-state current with low subthreshold swing at low operating voltage. Additionally, the heterogate dielectric and dual-electrode structure significantly enhance the device’s DC and RF performances, making the proposed HDL-TFET highly promising for low-power applications.

## 2. Device Structure and Model

**Figure 1:**
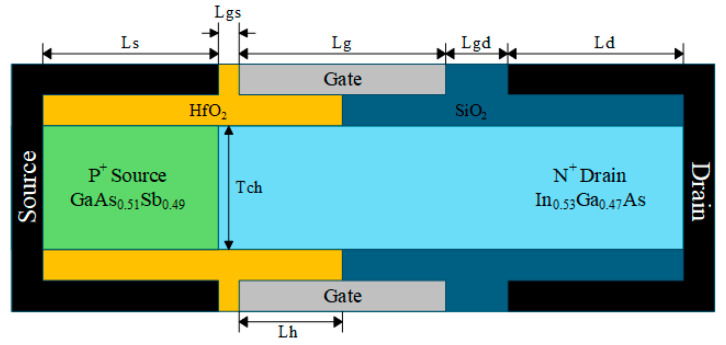
HDL-TFET device structure.

The device structure of the HDL-TFET is illustrated in Figure 1. The tunneling junction consists of a P^+^ GaAs_0.51_Sb_0.49_ source region and an intrinsic In_0.53_Ga_0.47_As channel region. The In_0.53_Ga_0.47_As/GaAs_0.51_Sb_0.49_ heterojunction epitaxial layer is lattice-matched to the InP substrate [26], which helps reduce the defect density at the heterojunction interface and maintains the device’s excellent off-state performance. Therefore, the material composition adopted in this work is In_0.53_Ga_0.47_As/GaAs_0.51_Sb_0.49_. Meanwhile, the HDL-TFET adopts a dual-electrode and heterogate dielectric structure, achieving outstanding DC and RF performances while suppressing off-state tunneling and reducing the impact of ambipolar current.

Relevant structural parameters are listed in Table 1. Based on the principle of charge plasma, the source work function is set to 5.0 eV to induce a high concentration of holes in the source region, forming an equivalent heavily doped P^+^ source. The drain work function is set to 4.4 eV to induce a low concentration of electrons in the drain region, forming an equivalent lightly doped N^+^ drain. The gate-source length (L_gs_) is 5 nm, and the gate-drain length (L_gd_) is 15 nm. The larger L_gd_ and lightly doped N^+^ drain are beneficial for achieving lower ambipolar current.

**Figure 2:**
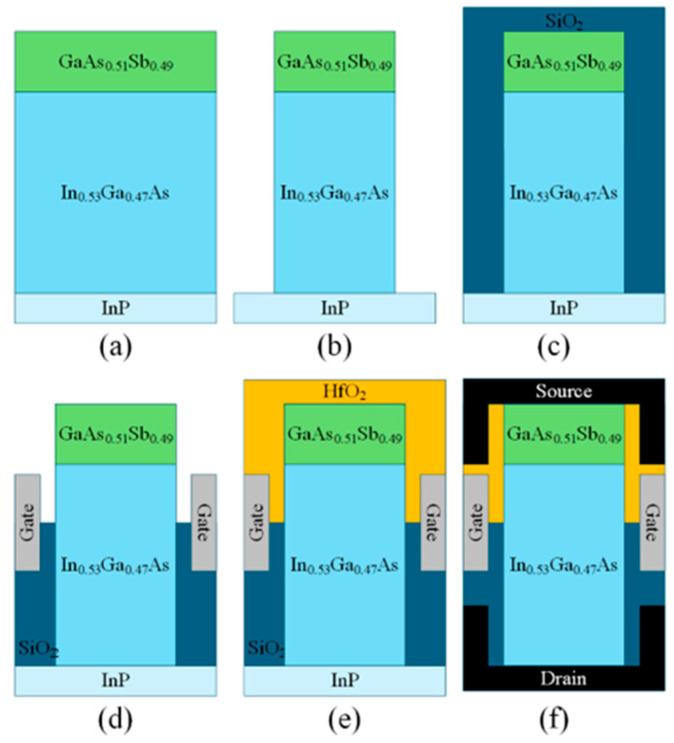
HDL-TFET fabrication process (**a**) epitaxial growth. (**b**) ICP etching. (**c**) SiO_2_ Deposition. (**d**) Etch SiO_2_ and deposit the gate electrode. (**e**) HfO_2_ Deposition (**f**) Form the source and drain electrodes.

Figure 2 illustrates the key fabrication process flow of the HDL-TFET. In Figure 2a, In_0.53_Ga_0.47_As and GaAs_0.51_Sb_0.49_ are epitaxially grown on the InP substrate. In Figure 2b, inductively coupled plasma (ICP) etching technology is used to form the GaAs_0.51_Sb_0.49_/ In_0.53_Ga_0.47_As heterojunction, which serves as the bulk material of the HDL-TFET. In Figure 2c, a SiO_2_ oxide layer is deposited via atomic layer deposition (ALD) technology. In Figure 2d, the SiO_2_ oxide layer is etched, and the gate electrode is deposited using ALD technology. In Figure 2e, a HfO_2_ oxide layer is deposited by ALD technology. In Figure 2f, the HfO_2_ oxide layer is first etched, followed by the deposition of the source metal in the etched region. The formation of the drain metal electrode can be accomplished via substrate lift-off technology, followed by etching of the SiO_2_ oxide layer and deposition of the drain metal, or alternatively via substrate thinning combined with via-hole technology [23], ultimately resulting in the device structure depicted in Figure 1.

Device simulations of the HDL-TFET were performed using the Silvaco ATLAS TCAD tool. To account for the spatial variation in energy bands, a non-local band-to-band tunneling model was employed in the simulations to more accurately simulate the tunneling process. The Lombardi mobility model, Fermi–Dirac carrier statistics, Shockley–Read–Hall recombination, band gap narrowing, and Auger recombination models were included in the simulations. Additionally, concentration-dependent and field-dependent mobility models, as well as trap-assisted tunneling models, were incorporated to provide simulation results that are closer to real-world behavior.

## 3. Results and Discussion

### 3.1. Operating Mechanism of the HDL-TFET

**Figure 3:**
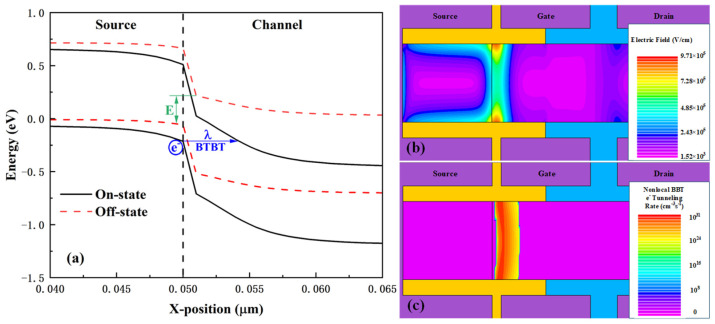
(**a**) Energy band diagram at 1 nm below the gate dielectric. (**b**) Electric field distribution in the on-state. (**c**) Electron tunneling rate distribution in the on-state.

Figure 3 illustrates the operating mechanism of the HDL-TFET. The energy band structures 1 nm below the gate dielectric near the tunneling junction are plotted in Figure 3a for both the off-state (V_ds_ = 0.5 V, V_gs_ = 0 V) and on-state (V_ds_ = 0.5 V, V_gs_ = 0.5 V) of the device. In the off-state, there is no overlap between the conduction band of the channel and the valence band of the source region near the tunneling junction, which suppresses BTBT. As Vgs increases, the conduction band edge of the channel drops below the valence band edge of the source region. This induces BTBT, where electrons tunnel from the source region to the channel, generating a tunneling current. In Figure 3a, E = 0.27 eV represents the energy difference between the conduction band and valence band at the tunneling junction in the off-state, while λ = 3.96 nm denotes the minimum tunneling distance at the tunneling junction in the on-state. Benefiting from the quasi-broken gap energy band structure of the In_0.53_Ga_0.47_As/GaAs_0.51_Sb_0.49_ heterojunction, both E and λ exhibit extremely small values. This indicates that compared with conventional tunneling junctions, the tunneling junction of the HDL-TFET is easier to turn on, allowing the device to operate at lower gate voltages while achieving higher tunneling currents.

From Figure 3a, it can also be observed that the conduction band and valence band of the source region exhibit a certain degree of downward shift in the on-state, which can be explained by Figure 3b. Figure 3b presents the electric field distribution in the bulk material during the on-state. It is evident that the electric field is mainly concentrated at the heterojunction, which is conducive to the HDL-TFET, achieving a larger on-state current. Meanwhile, the electric field shows a high distribution at the surface of the source region, which accounts for the downward shift in the source region’s conduction and valence bands. Figure 3c illustrates the distribution of the electron tunneling rate in the on-state. It is easy to see that due to the adoption of the heterojunction and dual-electrode symmetric structure, the device exhibits extremely high electron tunneling rates both at the surface and in the bulk along the vertical direction, even though it has a point tunneling structure. BTBT occurs across the entire heterojunction interface.

**Figure 4:**
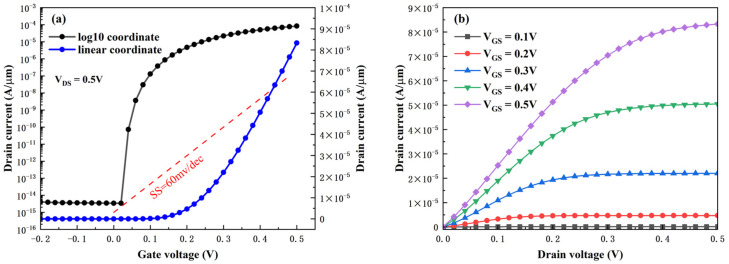
(**a**) Transfer characteristic, (**b**) output characteristic.

Figure 4a,b present the transfer characteristic curve and output characteristic curve of the HDL-TFET, respectively. Owing to the operating mechanism mentioned earlier, the device exhibits excellent DC performance. It can operate at a low voltage of 0.5 V, achieving a high I_ON_ up to 8.33 × 10^−5^ A/μm. Meanwhile, the heterogate dielectric and lightly doped N^+^ drain structure suppress off-state tunneling, resulting in an ultra-low I_OFF_ of 3.42 × 10^−15^ A/μm. No obvious ambipolar current is observed within a certain negative voltage range.

(1) $${SS}_{avg}=\frac{V_{th}-V_{off}}{{log(I}_{th})-{log(I}_{off})}$$

The average subthreshold swing (SS_avg_) of the device is calculated using Equation (1), where the voltage corresponding to a drain current (Ith) of 1 × 10^−7^ A/μm is taken as the threshold voltage (Vth). The resulting SS_avg_ is 10.18 mV/dec, which is far below the 60 mV/dec limit of MOSFETs.

### 3.2. Effect of Device Parameters on Performance

**Figure 5:**
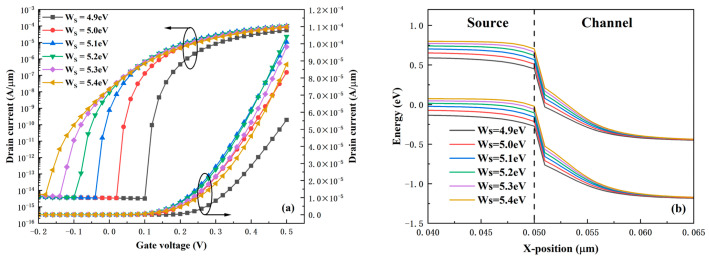
(**a**) Transfer characteristics under different source work functions. (**b**) On-state energy band diagrams under different source work functions.

Figure 5a shows the transfer characteristic curves of the HDL-TFET under different source work functions W_s_. It can be observed that the transfer curves shift gradually to the left as W_s_ increases. This phenomenon is easy to understand: with the increase of W_s_, the conduction band and valence band of the source region shift upward, leading to a lower turn-on voltage of the tunneling junction and easier formation of the BTBT path. Meanwhile, it is found that the subthreshold swing of the device increases with the increase of W_s_. The I_ON_ increases gradually when W_s_ is less than 5.2 eV, but starts to decrease when W_s_ exceeds 5.2 eV. As shown in Equation (2), based on the WKB approximation [27], the tunneling probability depends on the effective tunneling length λ and the effective tunneling window ∆Φ.

(2) $$T_{WKB}\approx exp(-\frac{4\lambda \sqrt{2m^{*}}\sqrt{E_{g}^{3}}}{3q\hbar (E_{g}+∆\Phi )})$$

From the on-state energy band diagram in Figure 5b, it can be observed that although the effective tunneling window ∆Φ increases with the rise in the W_s_, the effective tunneling length λ also increases as W_s_ increases. These two factors exert opposite effects on the tunneling probability. As a result, the variation in current is not as drastic as that of voltage, leading to the phenomena where the subthreshold swing increases and the I_ON_ first rises and then decreases.

**Figure 6:**
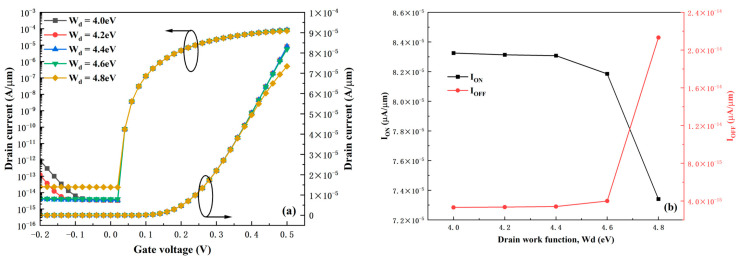
(**a**) Transfer characteristics under different drain work functions. (**b**) I_ON_ and I_OFF_ under different drain work functions.

Figure 6 presents the performance variations in the HDL-TFET under different drain work functions W_d_. The results indicate that the device is not sensitive to changes in W_d_. When W_d_ is less than 4.6 eV, there are no obvious changes in the I_ON_ and I_OFF_ of the device. The most significant change is that the ambipolar current decreases with the increase of W_d_—this is because the reverse tunneling window between the channel and the drain region reduces as W_d_ increases, lowering the reverse tunneling current. However, due to the heterogate dielectric structure, the variation in the ambipolar current is not drastic either. This phenomenon suggests that the device allows a wide range of fluctuations in W_d_ during the fabrication process, reducing the device’s manufacturing difficulty. It should be noted, however, that when W_d_ exceeds 4.6 eV, the on-state and off-state performances of the device degrade significantly. This is mainly attributed to the enhanced SRH recombination current in the off-state and the higher electron potential barrier in the on-state. Therefore, the optimal choice for W_d_ is 4.4 eV.

**Figure 7:**
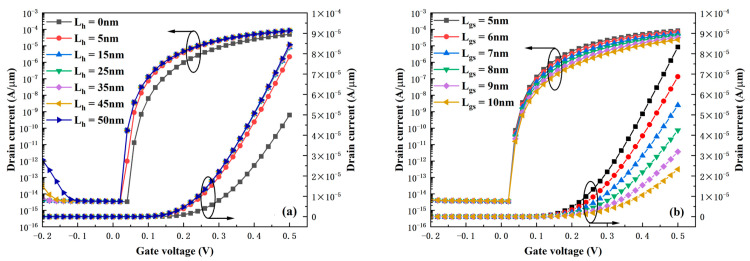
(**a**) Transfer characteristics under different HfO_2_ gate dielectric lengths L_h_. (**b**) Transfer characteristics under different gate-source length L_gs_.

The transfer characteristics of the HDL-TFET as a function of a HfO_2_ gate dielectric length L_h_ are presented in Figure 7a. Similarly to the variation in the drain work function W_d_, the device also exhibits robustness against changes in L_h_. It can be clearly observed from Figure 7a that as long as the variation of L_h_ does not affect the vicinity of the tunneling junctions between the source and channel, and between the drain and channel (L_h_ = 0 nm, L_h_ = 50 nm), the device performance remains essentially unchanged. This significantly reduces the manufacturing difficulty and improves the device reliability. Figure 7b presents the variation in transfer characteristics of the HDL-TFET under different gate-source lengths L_gs_. It can be clearly observed that the value of L_gs_ affects the device current under any positive voltage—with the increase of L_gs_, the current decreases accordingly. In the on-state, the I_ON_ when L_gs_ = 10 nm decreases by 72% compared with that when L_gs_ = 5 nm. This phenomenon is easy to understand: as L_gs_ increases, the band buffer region between the source and the gate becomes larger, while the energy level of the band under the corresponding voltage remains unchanged. This makes the band variation gentler and leads to a significant increase in the effective tunneling length λ, thereby severely degrading the device current.

The results indicate that the HDL-TFET exhibits a certain degree of stability against most process fluctuations. However, the key drawback lies in the significant impact of process variations at the heterojunction, making the process parameters at the heterojunction a critical focus during device fabrication.

### 3.3. Heterogate Dielectric Engineering

**Figure 8:**
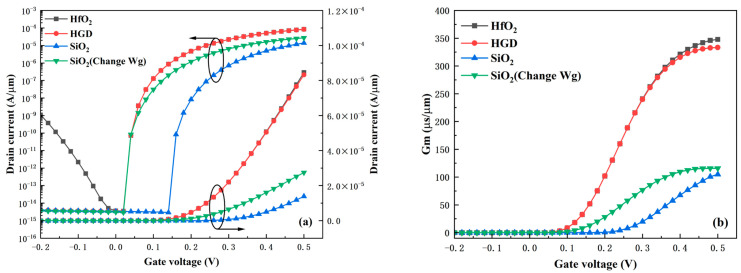
(**a**) Transfer characteristics and (**b**) transconductance G_m_ under different gate dielectrics.

An important feature of the HDL-TFET is the adoption of a heterogate dielectric (HGD) structure. This structure not only enhances the device’s DC performance but is also crucial for reducing parasitic capacitance, thereby greatly improving the device’s application value in high-frequency circuits. The primary advantage of the HGD structure lies in its ability to integrate the merits of different gate dielectrics for the independent optimization of each region, which is different from the stacked gate dielectric structure studied in Reference [28]. The stacked gate dielectric structure optimizes the device as a whole; the improvement in on-state performance means the degradation in off-state performance. Thus, the HGD structure is more conducive to the comprehensive enhancement of device performance. Figure 8a presents the transfer characteristics when the gate dielectric is HfO_2_, HGD, and SiO_2_, respectively. For the fairness of comparison, the transfer characteristic curve of the SiO_2_ gate dielectric device—with its tunneling turn-on voltage adjusted to be consistent with other cases by modifying the gate work function W_g_—is also plotted in Figure 8a. It can be observed that the HGD device inherits the low I_OFF_ of the SiO_2_ device and the high I_ON_ of the HfO_2_ device. Compared with the SiO_2_ device with adjusted W_g_, the I_ON_ increases from 2.75 × 10^−5^ A/μm to 8.33 × 10^−5^ A/μm. Compared with the HfO_2_ device, the ambipolar current decreases from 1.1 × 10^−9^ A/μm to 4.1 × 10^−15^ A/μm, a reduction of 6 orders of magnitude. Figure 8b shows the comparison of transconductance G_m_ under different gate dielectrics. G_m_ is defined as shown in Equation (3) and measures the device’s amplification capability. The results indicate that the G_m_ of the HGD structure is much larger than that of the SiO_2_ structure and basically consistent with that of the HfO_2_ structure. This endows the device with excellent amplification capability, which usually enables higher gain in circuit applications.

(3) $$G_{m}={dI}_{ds}/{dV}_{gs}$$

**Figure 9:**
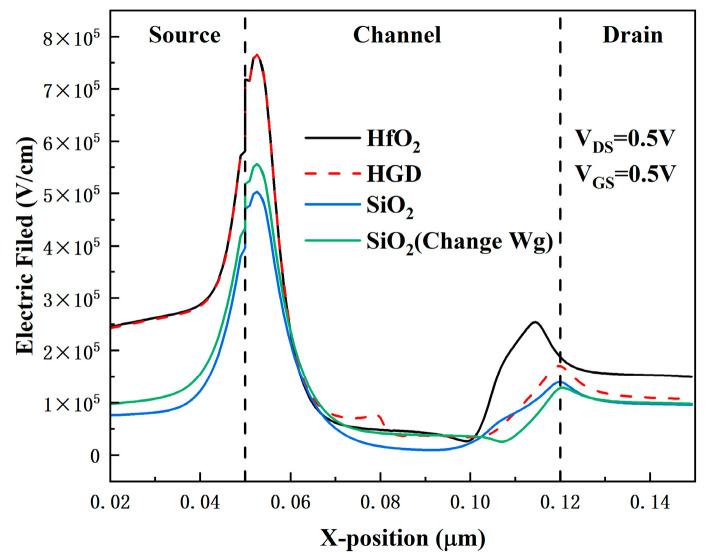
Electric field distributions at 1 nm beneath the gate dielectric in the on-state for different device structures.

The main reason for the performance improvement of the HGD structure lies in the variation in the electric field caused by the difference in dielectric constants between HfO_2_ and SiO_2_. Figure 9 presents the electric field distributions at 1 nm beneath the gate dielectric in the on-state for different device structures, which reveals the underlying mechanism responsible for the variations in the transfer curves shown in Figure 8a. At the gate-source region, the HGD structure and the HfO_2_ structure exhibit the strongest electric field, which significantly enhances I_ON_. In contrast, at the gate-drain region, the electric field of the HGD structure is lower than that of the HfO_2_ structure due to the low dielectric constant of SiO_2_—and this is the origin of the low leakage current. Additionally, the optimization of parasitic capacitance by the HGD structure is closely related to the electric field distribution shown in Figure 9.

**Figure 10:**
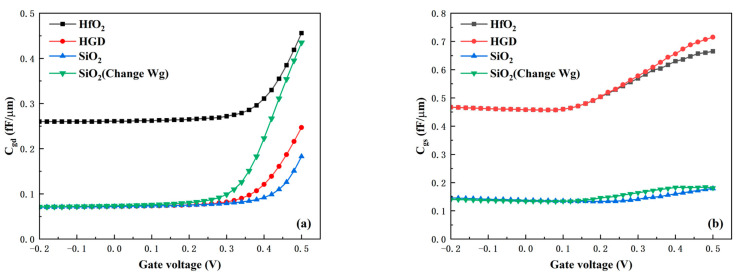
(**a**) Gate-drain capacitance C_gd_ and (**b**) gate-source capacitance C_gs_ under different gate dielectrics.

The optimization of parasitic capacitance by the HGD structure is demonstrated in Figure 10a,b. Figure 10a,b present the variations in gate-drain capacitance C_gd_ and gate-source capacitance C_gs_ under different gate dielectrics, respectively. It can be clearly observed that the C_gd_ of the HGD device is at the same level as that of the SiO_2_ device. Compared with the C_gd_ of the HfO_2_ device in the on-state, the HGD device’s C_gd_ decreases by 46%. However, the C_gs_ of the HGD device is at the same level as that of the HfO_2_ device and higher than that of the SiO_2_ device. This observation is consistent with the electric field distribution illustrated in Figure 9. Since C_gd_ depends on the electric field near the gate-drain region and C_gs_ is dependent on the electric field near the gate-source region, the HGD structure effectively reduces C_gd_ while causing no significant change in C_gs_. Even so, the maximum value of C_gs_ is only 0.71 fF. This C_gs_ value is completely acceptable given the device’s excellent DC performance and extremely low C_gd_. The HGD structure enables the device to have both high G_m_ and low parasitic capacitance, endowing it with excellent RF characteristics and high potential for high-frequency applications.

### 3.4. Optimization of the Dual-Electrode Structure

**Figure 11:**
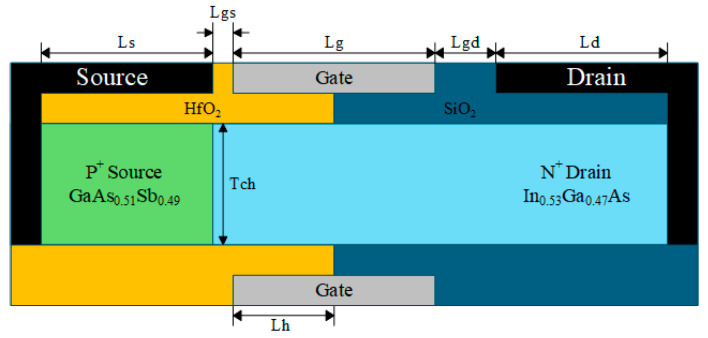
HDL-TFET with a single-electrode structure.

Figure 11 shows the HDL-TFET with a single-electrode structure (SE). Compared with the dual-electrode structured HDL-TFET (DE) proposed in this work, the SE structure can further simplify the fabrication process—it only requires depositing electrode metals via ALD technology. Although the DE structure increases process complexity compared with the SE structure, its improvement in device performance is noteworthy.

**Figure 12:**
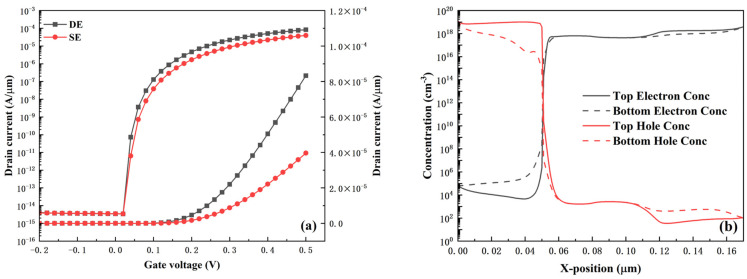
(**a**) Comparison of transfer characteristics between DE and SE structures. (**b**) Electron and hole concentration distributions at the top and bottom of the SE structure.

**Figure 13:**
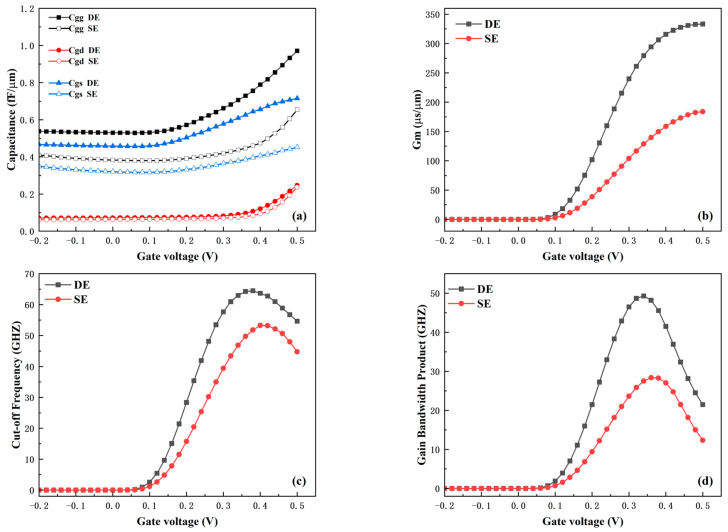
Comparisons of (**a**) parasitic capacitance, (**b**) transconductance G_m_, (**c**) cut-off frequency f_T_, and (**d**) gain bandwidth product GBP between DE and SE structures.

As shown in Figure 12, the performance enhancement of the DE structure over the SE structure, in terms of DC characteristics, mainly focuses on the subthreshold swing and on-state current. The SE structure exhibits an SS_avg_ of 13 mV/dec and an I_ON_ of 3.96 × 10^−5^ A/μm, which are 28% and 52% worse than those of the DE structure, respectively. Figure 12b illustrates the degradation mechanism of the SE structure: the carrier distribution induced near the top gate and bottom gate of the SE structure is inconsistent. The SE structure has no source-drain electrodes at the bottom, and the bulk material near the bottom gate is relatively far from the top electrode. This reduces the concentration of carriers induced in the bulk material near the bottom gate, ultimately degrading carrier tunneling around the bottom gate. However, as observed in Figure 3c, the electron tunneling rate distribution of the DE structure exhibits symmetry between the top gate and the bottom gate. The enhanced carrier tunneling near the bottom gate further optimizes the device performance. This is also reflected in the optimization of the device’s RF performance presented in Figure 13.

The RF characteristics of the device are crucial parameters for integrated circuit applications. Figure 13 presents a comparison of the RF parameters between the two electrode structures under a 1 MHz AC signal. Figure 13a shows the parasitic capacitance characteristics of the DE and SE structures. The results indicate that the DE structure has relatively larger parasitic capacitance, mainly because the introduction of the bottom source-drain electrodes increases the parasitic capacitance between the gate and drain, as well as between the gate and source. Although the parasitic capacitance performance of the DE structure is slightly degraded, the RF performance of the device requires a comprehensive evaluation. Figure 13b–d show the comparisons of transconductance (G_m_), cut-off frequency (f_T_), and gain-bandwidth product (GBP) between the two structures. These three parameters are calculated using Equations (3)–(5) [29].

(4) $$f_{T}=G_{m}/2\pi C_{gg}$$

(5) $$GBP=G_{m}/2\pi 10C_{gd}$$

G_m_ measures the amplification capability of the device; thus, the higher gain in circuit applications necessarily requires a larger G_m_. As shown in Figure 13b, benefiting from the optimization of the DE structure, the G_m_ of the DE device is significantly higher than that of the SE structure. The peak value of G_m_ reaches 333 μS/μm, which is an 81% improvement compared with the SE structure. According to Equations (4) and (5), f_T_ and GBP are proportional to G_m_ and inversely proportional to parasitic capacitance. Since the improvement in G_m_ brought by the DE structure is far greater than the degradation of parasitic capacitance, it can be observed from Figure 13c,d that the DE structure achieves higher f_T_ and GBP. The peak value of f_T_ is 64 GHz, which is a 21% increase compared with the SE structure; the peak value of GBP is 49 GHz, representing a 75% improvement over the SE structure. In summary, the introduction of the DE structure significantly enhances the DC and RF performance of the device.

To further demonstrate the advantages of the work presented in this paper, Table 2 summarizes the performance comparison between this work and other studies reported in the literature.

## 4. Conclusions

In this work, an In_0_._53_Ga_0_._47_As/GaAs_0_._51_Sb_0_._49_ heterojunction dopingless TFET with a heterogate dielectric is proposed and investigated, aiming to extend the advantages of dopingless TFET in low-power applications. Benefiting from the superiority of the quasi-broken gap energy band of the In_0_._53_Ga_0_._47_As/GaAs_0_._51_Sb_0_._49_ heterojunction, the HDL-TFET achieves extremely high BTBT efficiency. To further enhance the device’s DC and RF performance, a heterogate dielectric structure and a dual-electrode structure are introduced. Under the combined effect of these advantages, the HDL-TFET operates at a low voltage of 0.5 V, delivering a high I_ON_ of up to 8.33 × 10^−5^ A/μm and a steep average subthreshold swing of 10.18 mV/dec. Meanwhile, thanks to the HGD structure and low drain work function, off-state tunneling of the HDL-TFET is further suppressed, resulting in an ultra-low I_OFF_ of 3.42 × 10^−15^ A/μm and a high I_ON_/I_OFF_ of 2.44 × 10^10^, with no obvious ambipolar current. In addition, a comprehensive evaluation of the performance improvements brought by the HGD and DE structures is also conducted based on DC and RF characteristics. The results demonstrate that the introduction of these two structures significantly enhances the device’s DC and RF performance. Compared with other reported works in the literature, the proposed HDL-TFET exhibits distinct advantages. The research in this paper will lay the foundation for practical experiments on subsequent devices. The main limitation of the HDL-TFET lies in device fabrication, as the nanoscale process flow poses considerable challenges. However, with the advancement of nanotechnology, the HDL-TFET, leveraging the advantage of a dopingless structure, will exhibit tremendous potential in low-power circuit applications.

## Figures and Tables

**Table 1:** Main parameters of HDL-TFET.

Parameter Name	Symbol	Value
Oxide thickness	T_ox_	2 nm
Channel thickness	T_ch_	10 nm
Source length	L_s_	50 nm
Gate length	L_g_	50 nm
Drain length	L_d_	50 nm
HfO_2_ gate dielectric length	L_h_	25 nm
Gate-source length	L_gs_	5 nm
Gate-drain length	L_gd_	15 nm
Gate work function	W_g_	4.7 eV
Drain work function	W_d_	4.4 eV
Source work function	W_s_	5.0 eV

**Table 2:** Performance comparison between this work and other studies reported in the literature.

	I_ON_ (A/μm)	I_ON_/I_OFF_	$SS_{avg}$	f_T_ (GHz)	GBP (GHz)
This work (V_GS_ = 0.5 V)	8.33 × 10^−5^	2.44 × 10^10^	10.18	64	49
Ref. [7] (V_GS_ = 1.5 V)	1.1 × 10^−5^	1.1 × 10^12^	100	-	-
Ref. [8] (V_GS_ = 2.0 V)	1.36 × 10^−6^	1.47 × 10^11^	91	0.31	0.33
Ref. [21] (V_GS_ = 0.5 V)	1.2 × 10^−5^	-	227	-	-
Ref. [23] (V_GS_ = 0.6 V)	1.67 × 10^−5^	1.96 × 10^8^	36.6	13	5.23
Ref. [25] (V_GS_ = 0.6 V)	4.05 × 10^−5^	4.86 × 10^9^	20.3	71	12
Ref. [27] (V_GS_ = 1.0 V)	5.88 × 10^−5^	5.88 × 10^12^	18.2	5.04	1.29
Ref. [30] (V_GS_ = 1.0 V)	1.69 × 10^−5^	8.46 × 10^11^	31.38	36	-

## Data Availability

The original contributions presented in the study are included in the article; further inquiries can be directed to the corresponding author.
